# Factors associated with uptake of dual contraception among HIV-infected women in Bungoma County, Kenya: a cross-sectional study

**DOI:** 10.11604/pamj.supp.2017.28.1.9289

**Published:** 2017-11-02

**Authors:** Agnes Mideva Mulongo, Raphael Wekesa Lihana, Jane Githuku, Zeinab Gura, Simon Karanja

**Affiliations:** 1 Jomo Kenyatta University of Agriculture and Technology, Kenya; 2 Field Epidemiology and Laboratory Training Program, Ministry of Health, Kenya; 3 Kenya Medical Research Institute, Nairobi, Kenya

**Keywords:** Dual contraception, non-barrier contraceptives, elimination of mother to child transmission, HIV, Kenya

## Abstract

**Introduction:**

dual contraception, the use of non-barrier contraceptive method in combination with condoms, is an effective strategy in the elimination of mother-to-child transmission (eMTCT) of human immunodeficiency virus (HIV) and the achievement of zero new HIV infections. Despite its effectiveness, dual contraception use among HIV-infected women in Kenya remains low. We identified factors associated with dual contraceptive uptake in Bungoma County, Kenya.

**Methods:**

this was a facility-based cross-sectional study in eight hospitals in Bungoma County. We interviewed women using structured questionnaires. We calculated descriptive statistics about the womens’ baseline characteristics, examined the association between dual contraceptive use and other factors by calculating Odds Ratios (OR) and 95% Confidence Intervals (CI) and performed logistic regression.

**Results:**

we recruited 283 HIV-infected women.Among all enrolled women, 190 (67.1%) were aware of dual method and only 109 (38.5%) used dual contraception. The preferred dual pattern was male condom plus injectable contraceptive used by 53.2% of women (58/109). Among the 174 women who did not use dual contraception, 86 (49.4%) preferred using male condoms alone for contraception. Women were more likely to use dual contraception method if they were aware of dual contraception (AOR 12.2, 95% CI 4.7 – 31.7), used non-barrier contraceptives (AOR 9.8 95%; CI 4.5 – 21.3) and had disclosed their HIV status (AOR 7.1 95% CI 2.8 – 18.2) compared to those who did not.

**Conclusion:**

dual contraceptive prevalence was low. Advocacy on dual contraception as an approach to preventing vertical transmission of HIV should be escalated in order to improve its uptake.

## Introduction

Dual contraception is a cost-effective strategy for elimination of mother-to-child transmission (eMTCT) of human immunodeficiency virus (HIV) and preventing maternal morbidity and mortality due to unintended pregnancies [1,2]. Dual contraception is the simultaneous prevention of sexually transmitted infection (STIs) and unintended pregnancy through use of non-barrier contraceptive methods such as hormonal contraceptives or intra-uterine devices in combination with male or female condoms [1,2]. Dual methods can be offered in all HIV care settings or through active referral to Family Planning (FP) clinics. In 2013, there were 1.5 million (25%) women living with HIV globally who had given birth [3]. Unintended pregnancies accounted for 21.3% of new paediatric HIV infections [3,4]. Of the 240,000 children who acquired HIV through mother-to-child transmission (MTCT), 90% were from Sub-saharan Africa of whom 20% were from Kenya [4,5]. Unintended pregnancies among HIV-infected women contribute to poor maternal outcomes and HIV infection in the newborn [1,2]. Dual contraception effectively offers protection against STIs and HIV and prevents unintended pregnancies [1,6]. However, no single method is 100% effective at preventing both unintended pregnancies and STIs.

Although dual protection can be achieved by consistent condom use alone, studies have observed that typical use of male condoms for contraception yields a one-year cumulative incidence of 15 – 17% unintended pregnancies [7,8]. Non- barrier contraceptive methods are effective at pregnancy prevention but not STI protection [8]. Although the World Health Organisation (WHO) recommends that HIV-infected women use dual contraception to ensure that their intended pregnancies are planned and to prevent occurrence of STIs [5,9], low frequency of dual contraception has been demonstrated in several studies [10-12].

In many instances, HIV-infected women have an over-reliance on condoms, which don’t offer effective contraception, especially when used inconsistently [13-16]. Consistent and correct condom use is determined by both the male partner and the womens’ ability to negotiate for safer sex [17–19]. Dual contraception is likely to occur if both partners are concerned about unintended pregnancy and HIV or STI [20] and whether the HIV-positive partner has disclosed his or her HIV status [17,19]. The socio-ecological model illustrates that uptake of dual contraception among HIV-infected women is influenced by a complex interaction of many factors at individual, relational, community and structural levels [18,21]. Studies have found an inverse association between age, education level and the likelihood of dual method [18,21,22]. While some studies have observed that women with higher than primary education had increased odds of dual contraceptive use [10,18,22], another study found that women with tertiary education were less likely to practice dual contraception than those with primary education [23]. Elsewhere, dual contraceptive use has been reported among women who were educated and ± 35 years [6,16,24]. Relationship factors like communication between partners and partner approval can influence decisions on whether or not to continue dual method use [6,23,25]. In Kenya, dual contraception use is uncommon, as suggested from studies that have indicated that over-reliance on male condom, coupled with inconsistent use, has led to high levels of unintended pregnancies [26,27]. In addition, Kenya has had a steady increase in pregnancy rates among HIV-infected mothers [28]. In 2013, an estimated 37,276 (49.6%) pregnancies occurred in known HIV-infected women, of whom 10% were from Bungoma County [28]. Although dual contraceptive options are available and accessible at all Kenyan public health facilities to all women, HIV-infected women still experienced unintended pregnancies and 11.8% of 69,815 HIV- infected women who attended Prevention of Mother To child Transmission (PMTCT) of HIV clinics acquired STIs in 2014 [29]. Therefore, we estimated prevalence and identified factors associated with dual contraceptive use among HIV-infected women in Bungoma county, Kenya.

## Methods

We conducted across-sectional study among sexually active HIV-infected women aged 15–49 years who were attending follow-up clinics in eight sub-county health facilities in Bungoma County. Located in Western Kenya and bordering Uganda, Bungoma county is predominantly rural, with a 4.5% fertility rate andthe HIV prevalence was estimated to be 3.5% [29,30]. In 2014 the number of people living with HIV was 36,065, of whom 16% were children and 4.7% were pregnant women [28,30]. In 2013, the contraceptive prevalence rate was 38.5%, which is lower than the national prevalence of 46% [31,32].

### Sample size determination, sampling and participant recruitment

We estimated a sample size of 283 women would be required using Cochrane’s formula for a single proportion and estimatingthe proportion of HIV- infected women who used dual contraception was 24.3% [27], assuming a 95% confidence interval and 5% level of significance. Sampling was done in two stages. First, each of the eight health facilities was allocated a sample size proportional to workload that was determined four months prior to study period. At each study site, we recruited eligible women through systematic random sampling using the daily registration list as a sampling frame. A woman was considered elligible if she was HIV-infected, sexually active, aged between 15-49 years, and a resident of Bungoma county. Sexual activity was ascertained by asking the potential participants if they had been sexually active in the last three months prior to the study. Each eligible client attending HIV clinics in the selected facilities during the study period was assigned a number from the frame. The first subject was selected randomly and then every 6th subject was included in the study till the required sample was obtained at each site. In instanceswhen the eligible candidate refused, the next eligible client on the list was approached.

### Study variables

Dual contraceptive method was defined as use of primarily anon-barrier contraceptive method like hormonal or intrauterine contraceptive device (IUCD) together with male or female condom. The dependent variable was self-reported dual contraceptive use for three months preceeding the study. Independent variables included age, education, employment, marital status, dual contraceptive awareness, contraceptive practice, HIV status of partner, disclosure of HIV status and pregnancy intention. Information concerning STI was verified through individual patient files that provided record of clinical encounters.

We obtained written consent from the study participants who expressed willingness to participate in the study before the interviews. Parents or guardians who had accompanied subjects below 18 years were requested to provide written informed consent if the minor assented to participate in the study. Study approval was obtained from Kenyatta National Hospital (KNH) and University of Nairobi (UON) Ethical review committee (KNH-ERC/A/104). We also received permission to carry out the study from the County Director of Health in Bungoma.

### Data collection, management and analysis

We conducted face-to-face interviews using a pretested structured questionnaire to collect information on socio-demographic variables, preferences and factors associated with dual contraception. The pre-coded responses were entered in MS-excel database, cleaned and analyzed using EPI info version 7. We determined current contraceptive method use based on respondents’ answers to a question regarding “contraceptive method used at last intercourse in the past 3 months”. We calculated the prevalence of dual contraceptive use and evaluated the association of demographic and reproductive factors with dual-method use. Descriptive statistics were presented as frequencies, proportions and means in univariate analysis.

The Pearson chi-square test was used to compare differences between groups. We considered a p-value of < 0.05 as statistically significant. We calculated odds ratios (OR) and 95% confidence intervals (CI) for the assessment of factors associated with dual contraceptive use. We used logistic regression model to calculate adjusted OR and 95% CI for the assessment of independent factors associated with dual contraceptive use using stepwise forward method in which factors that were found significant in the bivariate analysis at P value 0.1 were selected and included into the final model.

## Results

### Sociodemographic profile of the participants

A total of 283 sexually active women were recruited into the study. Their mean age was 32 (±7) years, 73.5% (208/283) were married, out of whom 28.8% (60/208) were in polygamous relationships. More than half of the respondents reported 5-10 years as the age difference between their own age and their partner’s age (Table 1).

**Table 1:** socio demographic characteristics of HIV infected women attending HIV clinics in Bungoma County, 2015

Variables	Frequency (n = 283)	Percentage (%)
**Age**		
16 – 20	6	2.1
20 – 29	103	36.4
30 –39	120	42.4
40 - 49	54	19.1
**Marital status**		
Single	38	13.4
Married	208	73.5
Divorced / Separated	16	5.7
Widowed	21	7.4
**If married, type of union ( n= 208)**		
Monogamous	148	71.2
Polygamous	60	28.8
**Aware of their co-wife’s HIV status**		
Yes	16	26.7
No	44	73.3
**Highest educational status**		
Primary	135	47.7
Secondary	122	43.1
Tertiary	26	9.2
**Occupation**		
Employed	30	10.6
Self – employed	116	41.0
Unemployed	137	48.4
**Religion**		
Christian	250	88.3
Muslim	33	11.7
**Husband’s occupation (n = 208)**		
Employed	50	24.0
Self employed	57	27.4
Unemployed	101	48.6

### Fertility desires and intentions, unintended pregnancies and STIs

The median parity was 3 (range 0-11) and the median number of living children was 4 (range 1–10). More than three quarters (82.3%) of the respondents had either had an abortion or been pregnant after their HIV diagnosis. Even though 49.8% (141) of the respondents described their last pregnancy either as mis-timed or unintended, only 28.4% (40/141) were using condoms and 38.3% (54/141) reported no contraceptive use before their last pregnancy. Despite the fact that 58.0% (164/283) of the respondents reported not ever wanting a child, only 39.6% had ever discussed future child bearing intentions with their partners and 41.5% (68/164) were not using effective contraceptive methods for pregnancy prevention.

### Prevalence, awareness and preference of dual contraceptive use

Dual contraceptive prevalence in this population was 38.5% (109/283) (Table 2). One hundred and thirty-six (71.6%) of the 190 respondents who were aware of dual methods reported that health providers were their main source of information. More than half (59.6%) of the 109 dual users were married women who were less than 35 years of age. The preferred dual pattern (53.2%) was male condom plus injectable contraceptive. Almost half (49.4%) of the women (86/174) who did not practice dual contraception used condoms only for prevention of pregnancy and STIs. Only 25.6% (30/117) of non-users of dual contraception who didn’t use non-barrier contraceptives were aware of emergency contraception pills (E-Pill) for prevention of unintended pregnancy. Reasons for not using the dual contraceptive included non-disclosure of ones’ HIV status 19.0% (33/174) and partner disapproval 17.2% (30/174).

**Table 2:** dual contraceptive prevalence, practice and preference among HIV positive women in Bungoma County, 2015

	Variables	Frequency	Percentage (%)
1	**Family Planning used before HIV diagnosis**		
	Yes	152	53.7
	No	131	46.3
2	**Method used (n = 152)**		
	Injectable	82	53.9
	Pills	56	36.8
	Implants	11	7.2
	Male Condoms	2	1.3
	Female Condoms	1	1.3
3	**Awareness of dual methods**		
	Yes	190	67.1
	No	93	32.9
4	**Source of dual method information (n = 190)**		
	Peers/ support group	54	28.4
	Health worker	136	71.6
5	**Dual contraception use**		
	Yes	109	38.5
	No	174	61.5
6	**Contraceptive preferrence and pattern**		
	Dual users (n = 109)		
	Male condoms & Depo Provera	58	51.4
	Male condoms &Implants	42	38.5
	Male condoms & Pills	6	5.5
	Male condoms & BTL	3	2.8
	Male condoms & IUCD	2	1.8
7	**Non dual users ( n = 174)**		
	Male condoms	86	49.4
	Injectable	34	19.4
	Implants	17	9.8
	Pills	5	2.9
	BTL	1	0.7
	No contraceptive use	31	17.8
8	**Reasons for non – use of dual methods (174)**		
	One method effective	54	31.0
	Non- disclosure	33	19.0
	Partner declines / disapproval	30	17.2
	No regular partner	17	16.0
	Desire for pregnancy	18	9.8
	Religious beliefs	2	1.1

Key: FP – Family Plannig: CD – Condoms: BTL – Bilateral Tubal Ligation; IUCD – Intra Uterine Contraceptive Decice

Thirty (10.6%) of the respondents had multiple sexual relationships in the past three months preceding the study. Of those who engaged in multiple sexual relationships, 40.0% practiced the dual method, 33.3% used hormonal contraceptives,16.7% used condoms, while 10.0% didn’t use any contraception. Most, 72.4% (205/283) of the respondents had received contraceptive counselling, of whom 78.1% (160/205) had been counselled in Prevention of Mother-To-Child Transmission (PMTCT) of HIV clinic, half of them 52.2% (107/205) had been counselled on advantages of dual method use and only 31.7% (65/205) had been counselled with their sexual partners. Although most (98.6%, 279/283) of the respondents had been told about condom use, only 37.3% had been shown how to use both male and female condoms and only 9.8% had ever used female condoms. Three quarters 73.4% (80/109) of those who practiced dual contraceptionbelonged to a support group that discussed the importance of dual method use.

Only half, (51.2%, 145/283) of the respondents had discussed dual contraceptive use with their sexual partners. Of the 138 respondents who had not discussed dual contaceptive use with their partners, lack of regular sexual partner (38.4%), uncooperative partner (29.7%) and non-disclosure of ones’ HIV status (14.5%) were the main reasons for not discussing dual use with partner.

### HIV status disclosure, knowledge of partner HIV serostatus and history of STIs

Three-quarters (212/283) of women had disclosed their positive HIV status to their partners. However, only 62.2% (176/283) knew the HIV status of their partners and 53 (30.1%) of these 176 women were in serodiscordant relationships. The most common reason for unknown HIV status of partner was avoidance of the partner to undergo HIV testing, given by 28 (26.2%) of 107 women who were unaware of the HIV status of their sexual partners. Only 16 (26.7%) of the 60 women in polygamous relationships were aware of the HIV status of their co-wives. Only 90 (31.8%) of all283 respondents were confident that they could refuse sexual intercourse if a condom was unavailable. From medical records review, 30 women (10.6%) of all 283 women had STIs in the past one year before the study. The STIs included syphilis 40.0% (12/30), gonorrhoea 33.3% (10/30), genital ulcer disease 16.7% (5/30) and 10%(3/30) trichomoniasis. None of the women had been diagnosed as being co-infected with two or more STIs.

### Factors associated with dual contraceptive use among HIV positive women

Women who were aware of dual contraception had 14.2 greater odds of using dual method (OR,114.2, 95% CI, 6.2 - 32.2) than those who were unaware. Women who received HIV and FP counselling in PMTCT clinics were more likely to use dual contraception than those counselled elsewhere (OR, 3.5; 95% CI, 1.7 – 7.3). Women who were counselled on all FP methods had 13.4 greater odds of dual contraceptive use (OR, 13.4, 95% CI, 4.7 - 38.4) than those counselled on some FP methods. Women who belonged to a psychosocial support group were more likely to use dual contraception (OR 2.0, 95% CI, 1.3 – 3.3) and those who usednon-barrier contraceptive methods had 14.6 greater odds of dual contraceptive use (OR, 14.6, 95% CI, 6.8 – 28.7) (Table 3). In multivariable analyses, knowledge of dual contraception as a form of pregnancy prevention and safer sex was associated with dual use (adjusted odds ratio (AOR), 12.2 95% CI, 4.7 – 31.7). Use of non- barrier contraceptives (AOR 9.8, 95% CI 4.5 – 21.3) and disclosure of one’s HIV status to a sexual partner (AOR 7.1, 95% CI, 2.8 – 18.2 ) were independently associated with dual contraceptive use (Table 4).

**Table 3:** bivariate and multivariate analysis of factors associated with dual contraceptive use among HIV positive women in Bungoma County, 2015

Variable	Dual use		OR (95%CI)	P Value	AOR (95%CI	P Value
	Yes	No				
**Education status**						
Post primary	66	82	1.7(1.1 – 2.8)	0.04	0.7 (0.4 -1.4)	0.3
Primary	43	92				
**Marital status**						
Never Married	3	35	0.1 (0.03 – 0.4)	0.0006	2.1 (0.4-8.9)	0.3
Ever Married	106	139				
**Marriage type**						
Polygamous	13	47	0.3 (0.2 – 0.7)	0.004	2.3 (1-5.6)	0.6
Monogamous	64	84				
**FP use prior to HIV diagnosis**						
Yes	73	79	2.4(1.5 – 4.0)	0.0004	1.2 (0.6 -2.4)	0.5
No	36	95				
**Disclosed HIV status**						
Yes	102	110	8.4(3.7 – 19)	< 0.001	7.1 (2.8-18.2)	0.001
No	7	64				
**Discussed pregnancy desires with partner**						
Yes	53	59	1.8(1.1 – 3.0)	0.02	1.2 (0.6 -2.4)	0.5
No	56	115				
**Counselled on all available FP methods**						
Yes	104	101	13.4 (4.7– 38.4)	< 0.001	3.4 (4.1 -14.6)	0.04
No	4	52				
**Place counselled**						
CCC/PSC	12	33	3.5 (1.7 – 7.3)	0.0003	1.3 (0.9 – 3.4)	0.6
PMTCT	92	68				
Non -barrier contraceptive use						
Yes	99	72	14.6(6.8 – 28.7)	0.001	9.8 (4.5 -21.3)	0.001
No	10	102				
**Dual contraceptive knowledge**						
Yes	102	88	14.2 (6.2-32.2)	0.001	12.2 (3.9-21.8)	0.0001
No	6	87				
**Belongs to psychosocial support group**						
Yes	59	65	2.0 (1.3 -3.3)	0.006	0.8 (0.4 – 1.6)	0.6
No	50	108				

KEY : FP – Family planning; HIV- Human Immunodeficiency Virus; PMTCT- Prevention of Mother To Child Transmission of HIV ; CCC- Comprehensive Care Centre; PSC – Patient Support Centre

**Table 4:** significant factors associated with dual contraceptive use among HIV positive women in Bungoma County, 2015

Variable	OR (95%CI)	P Value	AOR (95%CI	P Value
**Disclosed HIV status**				
Yes	8.4(3.7 – 19)	< 0.001	7.1 (2.8-18.2)	0.001
No				
**Counselled on all available FP methods**				
Yes	13.4 (4.7– 38.4)	< 0.001	3.4 (4.1 -14.6)	0.04
No				
**Non -barrier contraceptive use**				
Yes	14.6(6.8 – 28.7)	0.001	9.8 (4.5 -21.3)	0.001
No				
**Dual contraceptive knowledge**				
Yes	14.2 (6.2-32.2)	0.001	12.2 (3.9-21.8)	0.0001
No				

## Discussion

Dual contraceptive use among HIV-infected women in Bungoma County, Kenya was associated with having knowledge on dual contraception, using non-barrier contraceptives and disclosing one’s HIV status to a sexual partner. Half of the respondents attending PMTCT clinics had been counselled on advantages of dual contraceptive use. Dual contraceptive use might be influencedby the high proportion (69.9%) of respondents inseroconcordant relationships, becausethey might less likely use condoms to prevent transmission of HIV. Our findingsare in the context of a county with high rates of unintended pregnancy, MTCTof HIVand STI transmission. The 38.5% prevalence of dual-contraceptive use among HIV-infected women in our study was higher compared to similar studies in Nigeria (27.2%), Uganda (3.5%) and India (23%) [1,33,34], while a study in Zimbabwe reported a similar proportion (38%) of dual contraceptive use [35]. However our prevalence of dual method usewas lower than that reported among women in Ethiopia (59.9%), Nigeria (45%) and the United States of America (47%) [10,36,37]. The high dual contraceptive prevalence in Ibadan, Nigeria may have been as a result of vigorous campaigns to scale-up dual contraception use by involving service providers and change in dual-protection counseling [36]. The high prevalence in USA could be due to high quality and strong integration of Sexual Reproductive Health (SRH) services withHIV services [37]. In Ethiopia, high uptake of could have been due to high injectables and condom use rates, with health provider advice being the main reason for dual method use [10]. Knowledge about dual contraception as a means of safer sex and birth control was strongly associated with dual method use in our study. Information on dual contraception was obtained during interactions with health providers and peer counsellors or mentor-mothers at PMTCT clinic visits and support groups. Similar findings were reported in South Africa, where knowledge about condoms was associated with condom use [38] and Nigeria, where dual contraceptive use was determined by the level of awareness on dual method. However, other studies have reported thatsocio-cultural factors influenced whether or not HIV-infected women would practice dual contraception despite being aware of the importance of dual contraception [39,40].

In this study, those women who were ever married were more likely to use dual methods. This might be because married partners find it easier to discuss issues regarding contraception than unmarried partners. Partner disapproval and non-discussion with partner were among the reasons for non-use of dual methods. Moreover, half of the dual method users belonged to a psychosocial support group where dual method use was highly advocated. Hence, there is evidence that women do not make decisions to use contraceptives unilaterally, but in consultation with their social networks who influence dual contraceptive use [41,42].

Use non-barriercontraceptiveswas associated with dual method use and the most preferred dual combination was injectable contraceptives andmale condoms. This might be because more than half of the respondents were using hormonal contraceptives before their HIV diagnosis, making it easy to incorporate condom use in their sexual life. A study in India documented that nonuse of modern contraceptives with increased focus on condoms alone resulted in low uptake of dual method use [15]. Other similar studies show a higher hormonal contraceptive uptake among HIV-infected women on dual methods [42,43]. We suspect that since non-barrier methods are mainly women controlled, this may be the reason for high dual method use. However, other studies reported decrease in condom use among women who used modern contraceptives [18,44].

Our study found that disclosure of one’s HIV status to a sexual partner was associated with dual method use. Disclosure likely facilitates open communication between partners in relation to HIV infection statussuch that both parties understand the importance of consistent dual contraceptive use and support each other in their efforts to prevent transmission or reinfection of HIV. Our finding was similar to findings from India and Zambia [15,34,44]. However, HIV status disclosure to regular partners was not associated with contraceptive use in Ethiopia [10].

This study revealed that even though many HIV-infected women do not desire future pregnancy, they still did not practice dual contraception as a means of safer sex and birth control. Similarly, a study in Uganda showed that women who didn´t disclose their HIV status to sexual partners and women who didn´t discuss on fertility issues were less likely to use contraceptives [45]. Psychosocial factors, like ability to negotiate for safe sex and discussion with sexual partner on dual use and pregnancy intentions can influence utilization of contraceptive methods.

This study has several limitations. Our study was cross-sectional in nature and thus could not allow us to determine causality, since contraceptive use and HIV status were assessed at the same point in time. The study also relied heavily on self-reported perceptions and behavior, which could result in over reporting of dual method use because of pressure from health care providers and social networks to practice safer sex and birth control increasing risk of social desirability bias.

## Conclusion

We found low prevalence of dual contraceptive use in a rural county in Kenya with high HIV prevalence. Knowledge on dual contraceptive, non- barrier contraceptive use and disclosure of ones’ HIV status to a sexual partner were key factors associated with dual method uptake. Social networks might also play a vital role in determining use or non-use of dual methods. We recommend health care providers to further embrace provider-initiated counseling approaches in order to introduce dual contraceptive use to all couples during post-test counselling whether they are seroconcordant or serodiscordant. In addition, we advocate for voluntary counselling and testing for all partners in polygamous relationships. Messages regarding the importance of dual contraception for STI and pregnancy prevention should be reinforced in both PMTCT and Comprehensive Care Clinics throughout the course of HIV care. Women who use condoms alone should be advised to access emergency contraception. Documentation of dual-protection practice within health management information systems will help on policy implementation regarding dual method use.

### What is known about this topic

- Dual contraception is the best strategy for preventing unintended pregnancy and HIV /STI among women living with HIV;
- The prevalence of dual method use varies significantly from different populations;
- Dual contraception can be achieved by consisted use of highly effective pregnancy prevention method (Modern contraceptives) and male or female condom.

### What this study adds

- Dual method use is high among women attending PMTCT clinics compared to those those attending Comprehensive Care Centres (CCC);
- Women who use condoms alone are not aware of emergency contraceptives as a method of pregnancy prevention;
- Majority of the women in polygamous relationships aren’t aware of the HIV status of their co-wives.

## Competing interests

The authors declare no competing interest.

## References

[1] Lawani Lucy, Onyebuchi Azubuike, Iyoke Chukwuemeka (2014). Dual method use for protection of pregnancy and disease prevention among HIV-infected women in South East Nigeria. BMC Womens Health.

[2] Reynolds Heidi, Janowitz Barbara, Homan Rick, Johnson Laura (2006). The value of contraception to prevent perinatal HIV transmission. Sex Transm Dis.

[3] UNAIDS The Gap Report: children and pregnant women living with HIV.

[4] Unaids Global Report UNAIDS report on the global AIDS epidemic 2013.

[5] WHO Global Update on the Health Sector Response to HIV, 2014.

[6] Tsuyuki Kiyomi, Barbosa Regina, Pinho Adriana de Araujo (2013). Dual protection and dual methods in women living with HIV: the brazilian context. J Sex Transm Dis.

[7] Trussell James, Warner David, Hatcher Rober (2005). Condom slippage and breakage rates. Fam Plann Perspect.

[8] Kost Kathryn, Singh Susheela, Vaughan Barbara, Trussell James, Bankole Akinrinola (2008). Estimates of contraceptive failure from the 2002 National Survey of Family Growth. Contraception.

[9] UNAIDS UNFPA, WHO and UNAIDS: Position statement on condoms and the prevention of HIV, other sexually transmitted infections and unintended pregnancy.

[10] Berhane Yemane, Berhe Haftu, Abera Gerezgiher, Berhe Hailemariam (2013). Utilization of modern contraceptives among HIV positive reproductive age women in Tigray, Ethiopia: a cross sectional study. Int Sch Res Not.

[11] Jhangri Gian, Heys Jennifer, Alibhai Arif, Rubaale Tom, Kipp Walter (2012). Unmet need for effective family planning in HIV-infected individuals: results from a survey in rural Uganda. J Fam Plann Reprod Health Care.

[12] Homsy Jaco, Bunnell Rebecca, Moore David, King Rachel, Malamba Samuel, Nakityo Rose (2009). Reproductive intentions and outcomes among women on antiretroviral therapy in rural Uganda: A Prospective Cohort Study. PLoS One.

[13] Haddad Lisa, Feldacker Caryl, Jamieson Denise, Tweya Hannock, Cwiak Carrie, Chaweza Thomas (2015). Pregnancy prevention and condom use practices among HIV-infected women on antiretroviral therapy seeking family planning in Lilongwe, Malawi. PLoS One.

[14] Morroni Chelsea, Heartwell Stephen, Edwards Sharon, Zieman Mimi, Westhoff Carolyn (2014). The impact of oral contraceptive initiation on young women’s condom use in 3 American cities: missed opportunities for intervention. PLoS One.

[15] Joshi Beena, Velhal Gajanan, Chauhan Sanjay, Kulkarni Ragini, Begum Shahina (2015). Contraceptive use and unintended pregnancies among HIV-Infected women in Mumbai. Indian J Community Med.

[16] Beyeza-Kashesya Jolly, Kaharuza Frank, Mirembe Florence, Neema Stella, Ekstrom Anna Mia, Kulane Asli (2009). The dilemma of safe sex and having children: challenges facing HIV sero-discordant couples in Uganda. Afr Health Sci.

[17] Nakaie Naomi, Tuon Sovanna, Nozaki Ikuma, Yamaguchi Fuzuki, Sasaki Yuri, Kakimoto Kazuhiro (2014). Family planning practice and predictors of risk of inconsistent condom use among HIV-positive women on anti-retroviral therapy in Cambodia. BMC Public Health.

[18] Pazol Karen, Kramer Michael, Hogue Carol (2010). Condoms for dual protection: patterns of use with highly effective contraceptive methods. Public Health Rep.

[19] Heffron Renee, Were Edwin, Celum Connie, Mugo Nellly, Ngure Kenneth, Kiarie James (2010). A prospective study of contraceptive use among African Women in HIV-1 Serodiscordant partnerships. Sex Transm Dis.

[20] Melaku Yohannes, Zeleke Ejigu Gebeye (2014). Contraceptive utilization and associated factors among HIV positive women on chronic follow up care in Tigray Region, Northern Ethiopia: a cross sectional study. PLoS One.

[21] Eisenberg David, Allsworth Jenifer, Zhao Qiuhong, Peipert Jeffrey (2012). Correlates of dual-method contraceptive use: an analysis of the National Survey of Family Growth (2006-2008). Infect Dis Obstet Gynecol.

[22] Muyindike Winnie, Fatch Robin, Steinfield Rachel, Matthews Lynn, Musinguzi Nicholas, Emenyonu Nneka (2012). Contraceptive use and associated factors among women enrolling into HIV care in Southwestern Uganda. Infect Dis Obstet Gynecol.

[23] Asfaw Hussen Mekonnen, Gashe Fikre Enquselassie (2014). Contraceptive use and method preference among HIV positive women in Addis Ababa, Ethiopia: a cross sectional survey. BMC Public Health.

[24] Beyeza-Kashesya Jolly, Kaharuza Frank, Ekström Anna Mia, Neema Stella, Kulane Asli, Mirembe Florence (2011). To use or not to use a condom : a prospective cohort study comparing contraceptive practices among HIV-infected and HIV-negative youth in Uganda. BMC Infect Dis.

[25] Pack Robert, Li Xiaoming, Stanton Bonita, Cottrell Lesley (2011). Psychosocial correlates of dual methods for contraception and STI protection in urban adolescents. ISRN Obstet Gynecol.

[26] Antelman Gretchen, Medley Amy, Mbatia Redempta, Pals Sherri, Arthur Gilly, Haberlen Sabina (2015). Pregnancy desire and dual method contraceptive use among people living with HIV attending clinical care in Kenya, Namibia and Tanzania. J Fam Plann Reprod Health Care.

[27] Ngure Kenneth, Ng’ang’a Zipporah, Kimani Violet, Khamadi Samoel, Onchiri Frankline, Irungu Elizabeth (2012). Correlates of contraceptive use among HIVdiscordant couples in Kenya. African Popul Stud.

[28] National AIDS Control Council (2014). Kenya AIDS Response Progress Report Progress towards Zero.

[29] Ministry of Health Kenya AIDS indicator survey 2012.

[30] National AIDS Control Council, National AIDS and STI Control Programme Kenya HIV County profiles.

[31] Kenya National Bureau of Statistics (KNBS); ORC Macro (2010). Kenya Demographic and Health Survey 2008-09. Heal San Fr.

[32] NASCOP (2014). Kenya AIDS Indicator Survey 2012: Final Report. J Acquir Immune Defic Syndr.

[33] Heys Jennifer, Kipp Walter, Jhangri Gian, Alibhai Arif, Rubaale Tom (2009). Fertility desires and infection with the HIV: results from a survey in rural Uganda. AIDS.

[34] Chakrapani Venkatesan, Kershaw Trace, Shunmugam Murali, Newman Peter, Cornman Deborah, Dubrow Robert (2011). Prevalence of and barriers to dual-contraceptive methods use among married men and women living with HIV in India. Infect Dis Obstet Gynecol.

[35] Magwali Thulani, Steiner Markus, Toms Harold, Brown Joelle (2005). How are condoms used in a family planning setting: evidence from Zimbabwe. Cent Afr J Med.

[36] Adeokun Lawrence, Mantell Joanne, Weiss Eugene, Delano Grace Ebun, Jagha Temple, Olatoregun Jumoke (2002). Promoting Dual Protection Planning Clinics in Ibadan, Nigeria. Int Fam Plan Perspect.

[37] Wilson Tracey, Koenig Linda, Walter Emmanuel, Fernandez Isabel, Ethier Kathleen (2003). Dual contraceptive method use for pregnancy and disease prevention among HIV-infected and HIV-uninfected women: the importance of an event-level focus for promoting safer sexual behaviors. Sex Transm Dis.

[38] Somera Yashpal, Ross Andrew (2013). Contraceptive knowledge and practice among HIV-positive women receiving antiretroviral therapy at a district hospital in KwaZulu-Natal. South African Fam Pract.

[39] Grabbe Kristina, Stephenson Rob, Vwalika Bellington, Ahmed Yusuf, Vwalika Cheswa, Chomba Elwyn (2009). Knowledge, use and concerns about contraceptive methods among sero-discordant couples in Rwanda and Zambia. J Womens Health (Larchmt).

[40] Tamene Wossenyelesh, Fantahun Mesganaw (2007). Fertility desire and family-planning demand among HIV-positive women and men undergoing antiretroviral treatment in Addis Ababa, Ethiopia. African J AIDS Res.

[41] Ochako Rhoune, Mbondo Mwende, Aloo Stephen, Kaimenyi Susan, Thompson Rachel, Temmerman Marleen (2015). Barriers to modern contraceptive methods uptake among young women in Kenya: a qualitative study. BMC Public Health.

[42] Behrman Jere, Kohler Hans -Peter, Watkins Susuan Cotts (2002). Social Networks and Changes in Contraceptive Use Over Time: Evidence From a Longitudinal Study in Rural Kenya. Demography.

[43] Keogh Sarah, Urassa Mark, Kumogola Yusuf, Mngara, Zaba Basia (2009). Reproductive behaviour and HIV status of antenatal clients in northern Tanzania: opportunities for family planning and preventing mother-to-child transmission integration. AIDS.

[44] Chibwesha Clara, Li Michelle, Matoba Christine, Mbewe Reuben, Chi Benjamin, Stringer Jeffrey Modern contraceptive and dual method use among HIV-Infected women in Lusaka, Zambia. Infect Dis Obstet Gynecol.

[45] Wanyenze Rhoda, Matovu Joseph, Kamya Moses, Tumwesigye Nazarius, Nannyonga Maria, Wagner Glenn (2015). Fertility desires and unmet need for family planning among HIV infected individuals in two HIV clinics with differing models of family planning service delivery. BMC Womens Health.

